# Chitosan-based infected wound care: A bibliometric analysis from 2002 to 2025

**DOI:** 10.1097/MD.0000000000047180

**Published:** 2026-01-23

**Authors:** Lan-Jing Zhang, Zou-Zou Yu, Dong-Dan Wang, Yue Shang, Ying-Ying Liu, Hui-Wen Zhang

**Affiliations:** a International Medical Services, Plastic Surgery Hospital, Peking Union Medical College, Chinese Academy of Medical Sciences, Beijing, China; b The Scar and Wound Treatment Center, Plastic Surgery Hospital, Peking Union Medical College, Chinese Academy of Medical Sciences, Beijing, China.

**Keywords:** bibliometric analysis, chitosan, drug delivery systems, hydrogel, wound infection

## Abstract

**Background:**

Chitosan has been increasingly used in the management of infective wounds over the past 2 decades. However, there is still a lack of comprehensive bibliometric analysis in this area. This study seeks to assess the global research landscape of chitosan in infected wound care, aiming to identify current trends and potential research directions.

**Methods:**

Data were retrieved from the Web of Science Core Collection and analyzed using VOSviewer, CiteSpace, and ArcGIS for bibliometric visualization. This analysis encompassed annual publication trends, key journals, authors, institutions, countries, and keywords.

**Results:**

This bibliometric analysis reviewed 501 publications (January 2002–April 2025), revealing a steady increase in research output. The *International Journal of Biological Macromolecules* published the most articles, while the *Chemical Engineering Journal* had the highest citation impact per article. China led in publication volume, followed by India and Iran. Institutional analysis identified the top 5 research centers, primarily based in China and Iran, with Qingdao University at the forefront. Among authors, Jayakumar Rangasamy was the most prolific, while Guo Baolin received the highest citations per article. Keyword analysis identified 2 main research areas: functional chitosan-based hydrogels and chitosan-based delivery systems for treating infective wounds, a globally significant healthcare challenge.

**Conclusions:**

Chitosan-based nursing in infected wound care is an increasingly important and clinically relevant option. Bibliometric analysis identified China, India, and Iran as the leading contributors to this field. Emerging research focuses on chitosan delivery system and chitosan mixed metal oxide nanoparticle antimicrobial agents in infected wound healing. The development of new chitosan-based technologies to combat wound infections and maintain efficacy against potential future drug-resistant bacteria will be essential for future advancements in wound care.

## 1. Introduction

The skin is a vital organ that covers the body’s surface, directly interacting with the external environment. It plays key roles in sensing external stimuli, regulating body temperature, and protecting the body from external harm.^[1]^ The disruption of the skin’s continuity may result in a wound. The incidence of chronic wounds such as diabetic foot ulcers and burn infections is on the rise worldwide.^[2]^ Notably, they are sometimes life-threatening complications and are associated with high mortality rates, significantly impacting the physical and mental well-being.^[3]^ They are characterized by difficult and delayed healing, both of which increase the risk of infection.^[4]^ Infections caused by antibiotic-resistant pathogens are not only challenging to treat but also lead to extended recovery times for patients and result in high healthcare costs.^[5]^ Antibiotic-resistant bacteria readily spreads in hospitals and frequently cause wound infections. The rise infections caused by multidrug-resistant bacteria (such as methicillin-resistant *Staphylococcus aureus* and vancomycin-resistant enterococci) creates a significant treatment dilemma. Inflammation is essential for wound healing, but excessive inflammation can cause tissue cell necrosis, hindering the healing process.^[6]^ As a result, various modern wound dressings, including semipermeable films, foams, hydrocolloids, and hydrogels with sustained drug release properties, have been developed to prevent infection in wound defects and enhance the healing process.^[7–10]^

Chitosan, a biopolymer derived from the alkaline deacetylation of chitin, comprises glucosamine and N-acetylglucosamine units. It is nontoxic, biocompatible, biodegradable, moisture retentive, and inexpensive. It can be chemically modified through methods including carboxymethylation, acylation, alkylation, and quaternization to synthesize a range of N-, O-, and N,O-modified chitosan derivatives.^[11]^ In managing excessive inflammation and chronic wound infections, chitosan-based wound-healing therapy have unique advantages.^[12,13]^ Chitosan-based self-healing hydrogels are reported to restore their original functionality after damage, thereby extending material lifespan, promoting wound closure, and preventing bacterial infections. As a result, chitosan may be one of the most effective options for treating infected wounds.

Bibliometric analysis uses large-scale quantitative methods to systematically collect and analyze metadata from research publications, a technique widely applied in plastic surgery research.^[14]^ However, to our knowledge, no bibliometric study has focused specifically on chitosan-based nursing in infected wound healing. To fill this gap, we conducted a bibliometric and visualization analysis of literature on chitosan nursing in infected wound healing, covering publications from 2002 to 2025. This study aims to map the development of chitosan nursing in this field, identify research hotspots, uncover emerging trends, consolidate existing knowledge, and promote innovation in the use of chitosan for infected wound healing.

## 2. Methods

### 2.1. Data collection

A systematic literature search was performed using the Web of Science Core Collection (WoSCC) database, covering publications from January 1, 2002, to May 9, 2025. All research procedures were completed within a single day. The search strategy aimed to capture relevant studies using the following query: TS=(“Chitosan” and “wound” and “infection”). Subsequently, to investigate common conjugates of chitosan and its various delivery systems, a search was conducted in the WoSCC using the query: TS=(“Chitosan” OR “Poliglusam”) AND TS=(“Nanoconjugates” OR “Nanoconjugate”). A total of 113 articles published between 2009 and 2025 were retrieved. This study only included English research articles and reviews; other publication types were excluded, including Letters (n = 1), Meeting Abstracts (n = 2), Proceeding Papers (n = 3), and Early Access articles (n = 3), resulting in 104 remaining articles. Among these, 95 were research articles and 9 were reviews. Two researchers independently screened the titles and abstracts of the retrieved articles. Discrepancies were resolved by a senior researcher. As no patient or animal was invovled in this research, ethical approval was not necessary.

### 2.2. Visualized analysis

**Figure 1.**
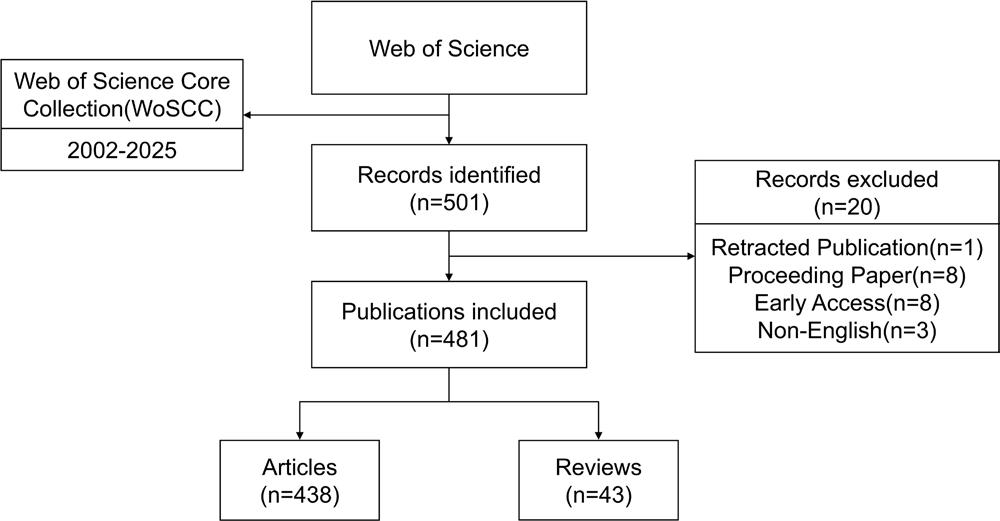
The workflow of data collection and processing procedures.

Bibliometric analysis was conducted with various analytical tools. The Science Citation Index Expanded (SCI-Expanded) was the primary data source, and trends in annual publications and citation frequencies were analyzed using Microsoft Excel 2021 (Microsoft, Washington). Collaborative networks among countries, authors, and institutions were visualized using VOSviewer 1.6.20, while ArcGIS 10.8 was used to display the geographical distribution of research output. Additionally, CiteSpace version 6.4.R1 （Philadelphia）was utilized for keyword co-occurrence analysis and detecting citation bursts in both keywords and references, revealing emerging research trends. Figure 1 outlined the data collection and processing workflow.

## 3. Results

A total of 501 articles published between January 1, 2002, and May 9, 2025, were initially identified. After applying inclusion and exclusion criteria, 20 articles were excluded, including retracted publications (n = 1), conference proceedings (n = 8), early access articles (n = 8), and non-English publications (n = 3). The final dataset comprised 481 articles, consisting of 438 research articles and 43 review articles.

### 3.1. Publication metrics

**Figure 2.**
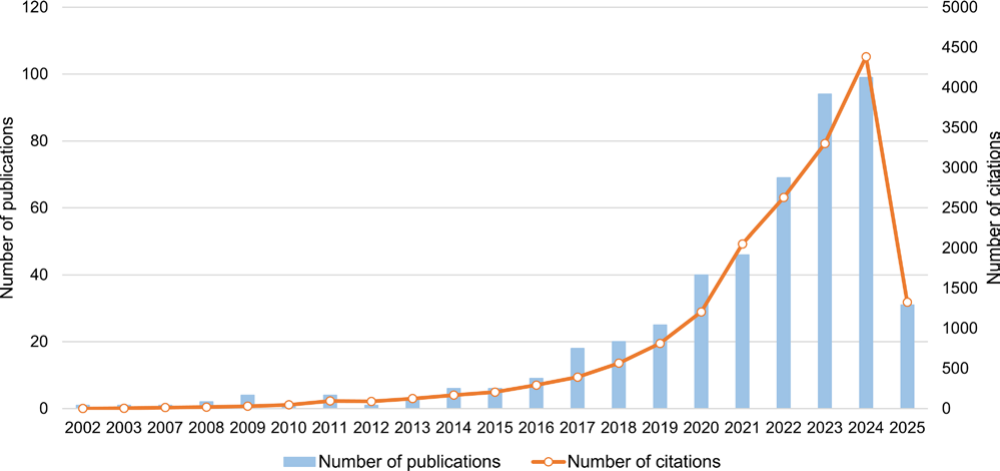
Annual publications and citation frequency pertaining to infected wound healing with chitosan-based wound care.

Research on chitosan-based nursing for infected wound healing has seen significant growth from 2002 to 2025, with publications steadily increasing from 1 in 2002 to a peak of 99 in 2024, particularly accelerating after 2017. Similarly, citations grew sharply from 1 in 2002 to 4384 in 2024 (Fig. 2). The marked rise in publications post-2017 corresponds with a substantial increase in citation frequency, indicating a concurrent surge in both research activity and academic interest in recent years. This overall trend suggests that the field is undergoing rapid development.

### 3.2. Leading journals and articles

**Figure 3.**
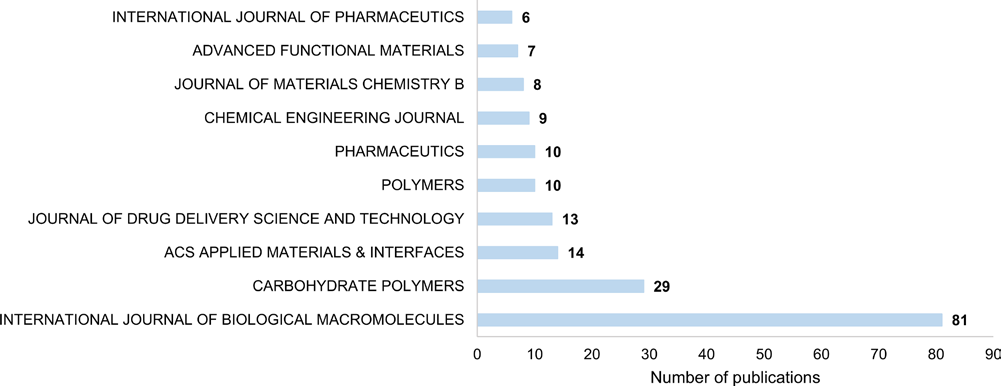
The top 10 journals ranked by publication volume.

Figure 3 shows the top 10 journals ranked by publication volume. The *International Journal of Biological Macromolecules* led with 81 publications and a citation/article (citation/article, c/a) ratio of 21.4. In comparison, the *Chemical Engineering Journal* had 9 publications but a much higher c/a ratio of 109.3, with a total of 984 citations. It was followed by the *International Journal of Pharmaceutics* (62.3 c/a) and *ACS Applied Materials & Interfaces* (60.71 c/a). Table 1 lists the top 10 most cited articles in this field.

**Table 1:** The top 10 most cited articles.

Rank	Title	First author	Corresponding author	Journal	Year	Total citations	Annual citations
1	Development of a chitosan-based wound dressing with improved hemostatic and antimicrobial properties.	Ong, Shin-Yeu	Lu, Jia	Biomaterials	2008	715	39.72
2	Chitosan preparations for wounds and burns: antimicrobial and wound-healing effects.	Dai, Tianhong	Hamblin, Michael R.	Expert Review of Anti-infective Therapy	2011	704	46.93
3	A novel wound dressing based on Ag/graphene polymer hydrogel: Effectively kill bacteria and accelerate wound healing.	Fan, Zengjie	Fan, Zengjie	Advanced Functional Materials	2014	687	57.25
4	A functional chitosan-based hydrogel as a wound dressing and drug delivery system in the treatment of wound healing.	Liu, He	Li, Zuhao	RSC Advances	2018	654	81.75
5	Degradable conductive injectable hydrogels as novel antibacterial, antioxidant wound dressings for wound healing.	Qu, Jin	Guo, Baolin	Chemical Engineering Journal	2019	597	85.29
6	A biomimetic Mussel-inspired ε-poly-L-lysine hydrogel with robust tissue-anchor and anti-infection capacity.	Wang, Rui	Xu, Hong	Advanced Functional Materials	2017	470	52.22
7	Antibacterial and angiogenic chitosan microneedle array patch for promoting wound healing.	Chi, Junjie	Wang, Yongan	Bioactive Materials	2020	354	59.00
8	Novel asymmetric wettable AgNPs/chitosan wound dressing: In vitro and in vivo evaluation.	Liang, Donghui	Chen, Rong	ACS Applied Materials & Interfaces	2016	346	34.60
9	Control of wound infections using a bilayer chitosan wound dressing with sustainable antibiotic delivery.	Mi, FL	Mi, FL	Journal of Biomedical Materials Research	2002	244	10.17
10	Germanene-based theranostic materials for surgical adjuvant treatment: Inhibiting tumor recurrence and wound infection.	Feng, Chan	Tao, Wei	Matter	2020	211	35.17

### 3.3. Distribution and collaboration of countries

**Figure 4.**
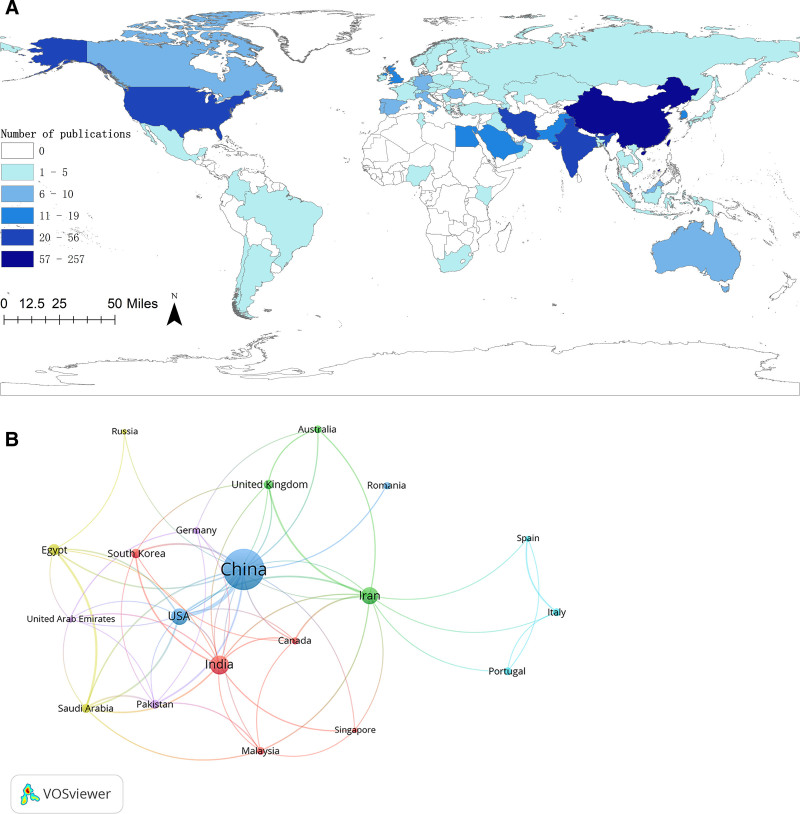
(A) The geographic distribution of publications. (B) Country collaboration analysis.

A total of 55 countries have contributed to this research area. Table 2 displays the publication output of the top 10 contributing countries. Figure 4A illustrates the geographic distribution of publications, with China leading at 257 publications (53.43%), followed by India (57, 11.85%) and Iran (44, 9.15%), collectively accounting for nearly 70% of global output. The country collaboration analysis, shown in Figure 4B, highlights China, the United States, and India as key players occupying central positions in the network.

**Table 2:** The publication output of the top 10 contributing countries.

Rank	Country	Count	% of 481	Citations	Average citations	*H*-index
1	China	257	53.43	11,034	42.93	48
2	India	57	11.85	1371	24.05	23
3	Iran	44	9.15	1261	28.66	20
4	USA	43	8.94	3536	82.23	27
5	Egypt	19	3.95	502	26.42	16
6	United Kingdom	15	3.12	462	30.80	9
7	South Korea	14	2.91	991	70.79	12
8	Saudi Arabia	13	2.70	285	21.92	7
9	Pakistan	13	2.70	247	19.00	8
10	Italy	10	2.08	549	54.90	8

### 3.4. Distribution and collaboration of institution

**Figure 5.**
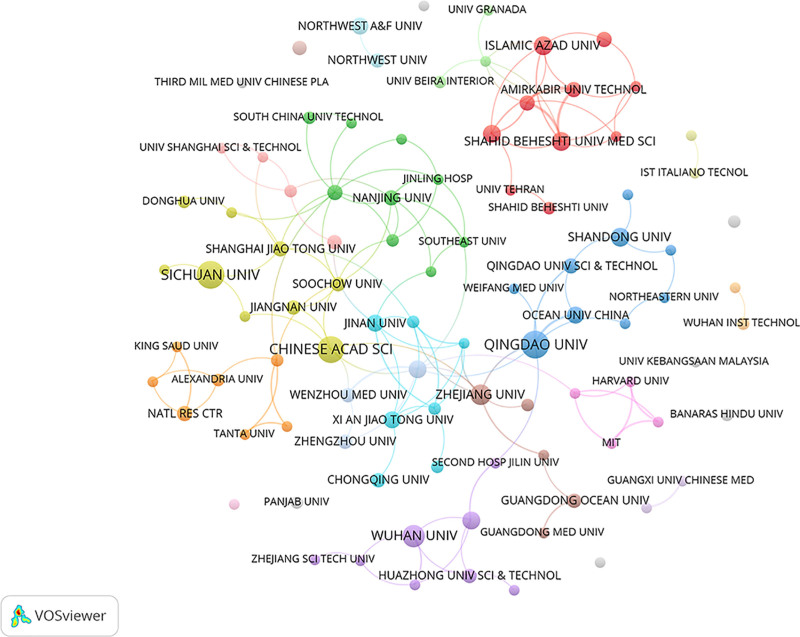
The collaboration network among institutions with ≥5 publications.

A total of 95 institutions have contributed to research in this field. Table 3 presents the top 10 institutions by publication volume. All of the top 10 institutions are located in China and Iran, including Qingdao University (19 publications, 3.95%), Sichuan University (18, 3.74%), Chinese Academy of Sciences (17, 3.53%), Wuhan University (12, 2.49%), Zhejiang University (10, 2.08%), Shahid Beheshti University of Medical Sciences (9, 1.87%), Shandong University (9, 1.87%), Jilin University (8, 1.66%), and University of the Chinese Academy of Sciences (8, 1.66%). Figure 5 shows the collaboration network among institutions with 5 or more publications.

**Table 3:** The top 10 institutions by publication volume.

Rank	Affiliations	Count	% of 481	Citations	Average citations	*H*-index
1	QINGDAO UNIV	19	3.95	494	26.00	12
2	SICHUAN UNIV	18	3.74	554	30.78	11
3	CHINESE ACAD SCI	17	3.53	1115	65.59	12
4	WUHAN UNIV	12	2.49	645	53.75	10
5	ZHEJIANG UNIV	10	2.08	585	58.50	6
6	SHAHID BEHESHTI UNIV MED SCI	9	1.87	263	29.22	7
7	ISLAMIC AZAD UNIV	9	1.87	228	25.33	8
8	SHANDONG UNIV	9	1.87	191	21.22	6
9	JILIN UNIV	8	1.66	805	100.63	6
10	UNIV CHINESE ACAD SCI	8	1.66	239	29.88	7

ACAD = academic, MED = medicine, SCI = sciences, UNIV = university.

### 3.5. Distribution and collaboration of author

**Figure 6.**
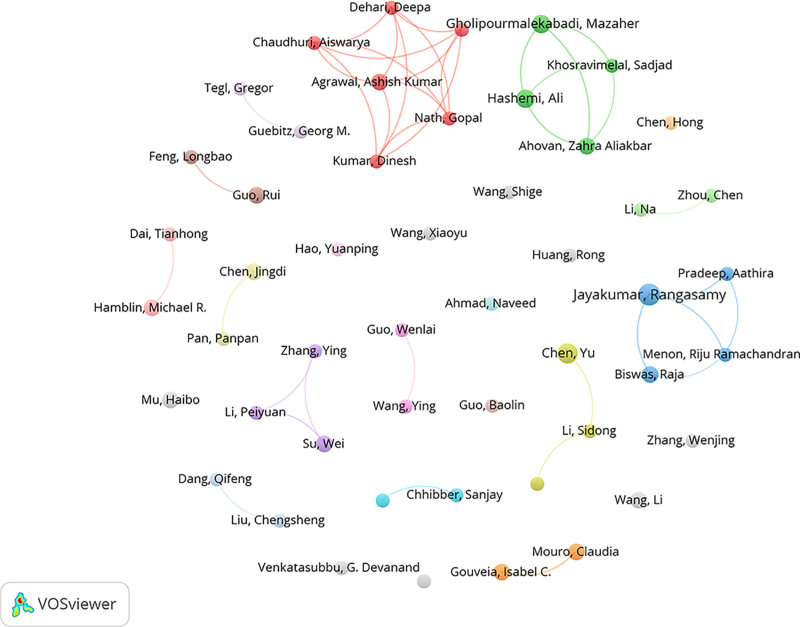
The cooperation network of authors.

A total of 3106 authors have contributed to this field. Table 4 presents the top 10 authors by publication volume and citation count. The most prolific authors include Jayakumar Rangasamy (7 publications, 1.46%), Chen Yu (6, 1.25%), Gholipourmalekabadi Mazaher (5, 1.04%), Hashemi Ali (5, 1.04%), Hamblin Michael R (4, 0.83%), Biswas Raja (4, 0.83%), Wang Li (4, 0.83%), Guo Rui (4, 0.83%), Gouveia Isabel C. (4, 0.83%), and Mouro Claudia (4, 0.83%). The most cited authors are Hamblin Michael R (952 citations, 238.00 c/a), Guo Baolin (939 citations, 313.00 c/a), Dai Tianhong (929 citations, 309.67 c/a), Jayakumar Rangasamy (209 citations, 29.86 c/a), Biswas Raja (196 citations, 49.00 c/a), Gholipourmalekabadi Mazaher (188 citations, 37.60 c/a), Wang Li (185 citations, 46.25 c/a), Venkatasubbu G. Devanand (179 citations, 59.67 c/a), Guo Rui (169 citations, 42.25 c/a), and Khosravimelal Sadjad (161 citations, 53.67 c/a). Figure 6 illustrates the author collaboration network.

**Table 4:** The top 10 authors by publication volume and citation count.

Rank	Author	Count	% of 481	Rank	Author	Citations	Average citations
1	Jayakumar, Rangasamy	7	1.46	1	Hamblin, Michael R.	952	238.00
2	Chen, Yu	6	1.25	2	Guo, Baolin	939	313.00
3	Gholipourmalekabadi, Mazaher	5	1.04	3	Dai, Tianhong	929	309.67
4	Hashemi, Ali	5	1.04	4	Jayakumar, Rangasamy	209	29.86
5	Hamblin, Michael R.	4	0.83	5	Biswas, Raja	196	49.00
6	Biswas, Raja	4	0.83	6	Gholipourmalekabadi, Mazaher	188	37.60
7	Wang, Li	4	0.83	7	Wang, Li	185	46.25
8	Guo, Rui	4	0.83	8	Venkatasubbu, G. Devanand	179	59.67
9	Gouveia, Isabel C.	4	0.83	9	Guo, Rui	169	42.25
10	Mouro, Claudia	4	0.83	10	Khosravimelal, Sadjad	161	53.67

### 3.6. Keywords analysis

**Figure 7.**
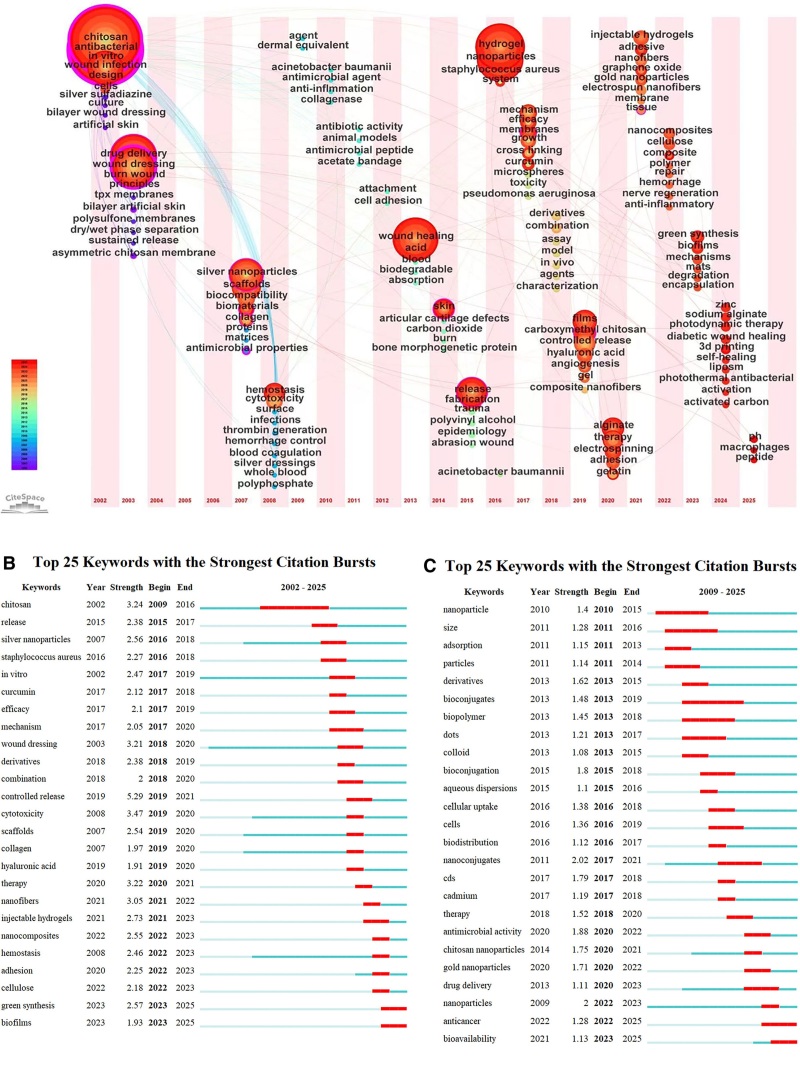
(A) The keyword timezone map. (B) The top 25 burst keywords of chitosan-based infected wound care. (C) The top 25 burst keywords of common conjugates of chitosan and its various delivery systems.

Figure 7A presents a keyword timezone map, where node size represents keyword frequency, and color (ranging from blue to red) indicates the time of appearance. Each node features concentric rings that reflect annual frequency, with wider bands representing higher frequency. Purple outer rings highlight keywords with centrality >0.1, underscoring their importance within the network. Lines between nodes indicate keyword co-occurrence, with thicker lines indicating stronger co-occurrence. Node labels identify keywords, and the *x*-axis displays the year each keyword 1st appeared. Figure 7B and C shows the top 25 burst keywords, revealing the evolution of research hotspots, trends, and potential future directions.

### 3.7. Co-citation analysis of references

**Figure 8.**
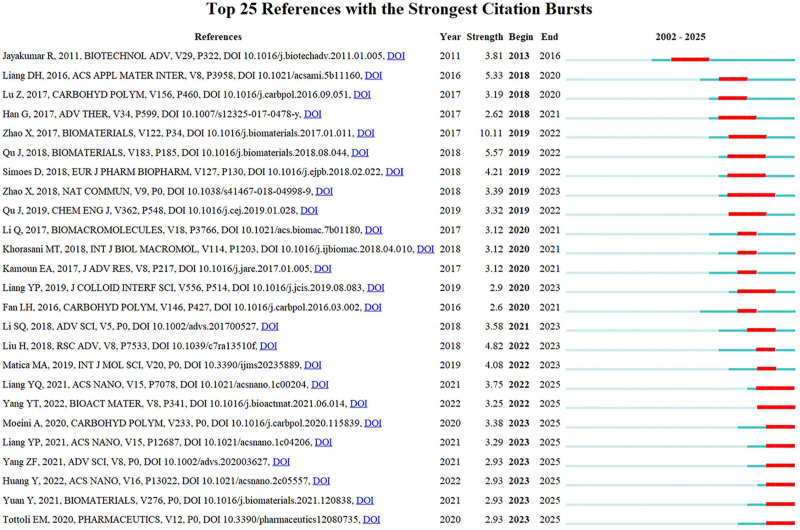
The top 25 references with the highest burst strength.

Burst analysis of references highlights rapidly emerging research areas, with stronger burst strength indicating greater influence during specific time periods. This analysis helps identify research frontiers and track the evolution of hotspots in the field. Figure 8 presents the top 25 references with the highest burst strength.

## 4. Discussion

Infected wound healing is a global challenge due to the critical functional and aesthetic roles of the skin.^[15]^ Wound care has become increasingly important and complex during the healing process, with bacterial resistance being a significant concern in modern medicine. Chitosan is widely used in infected wound care for its antibacterial properties, conductivity, anti-oxidation, anti-inflammatory effects, stimulus–response capabilities, adhesion, hemostasis, and controlled release functions. Recent advancements in chitosan-based wound research-particularly in water solubility, membrane permeability, and targeted drug delivery-have improved material stability and spurred a sharp rise in publications.^[16]^ The substantial volume of publications has provided healthcare workers with invaluable insights, while simultaneously posing a challenge in comprehensively evaluating the field and identifying its development trends and research hotspots. To our knowledge, no previous bibliometric study has been conducted on this topic. To address this, we conducted a bibliometric analysis using VOSviewer, CiteSpace, and ArcGIS on 501 articles from the WoSCC over the past 2 decades, representing, to our knowledge, the 1st such study in this domain.

Our analysis revealed distinct geographic and institutional patterns. China, India, and Iran collectively contributed nearly 70% of the global research output. However, although China led in publication volume, its average citation per article (c/a ratio) of 42.93 lagged behind that of the United States (82.23), suggesting differences in research impact. At the institutional level, contributions were relatively balanced, with Qingdao University emerging as the most prolific institution. Iranian institutions such as Shahid Beheshti University of Medical Sciences also played active roles. Collaboration among Chinese institutions was particularly strong, fostering technological innovation through dense internal networks. Nevertheless, the scholarly influence of individual authors revealed a notable disparity: while East Asian researchers dominated the top 10 by publication volume, only 2 ranked among the top 10 by citations. High-impact authors such as Zhu Songsong (c/a 36.14) and Khadka Ashish (c/a 30) achieved visibility through international collaborations. Overall, the collaboration network remains centered in East Asia, with limited connections to European and North American researchers, suggesting that future work would benefit from more interdisciplinary and cross-regional partnerships to enhance theoretical originality and global applicability.

During the initial phase (2010–2016), research efforts predominantly focused on fundamental materials science exploration. The emergence of keywords such as “nanoparticle” (Strength = 1.4), “size” (Strength = 1.28), and “adsorption” (Strength = 1.15) reflects a dedicated emphasis on optimizing and characterizing the physicochemical properties of nanoparticles themselves. Shortly thereafter, beginning around 2013, concepts such as “bioconjugates” (Strength = 1.48) and “biopolymer” (Strength = 1.45) began to gain prominence, signaling a shift in research perspective from mere material synthesis toward the preliminary construction of biocompatible systems, thereby laying a theoretical foundation for subsequent delivery system development. Between 2017 and 2021, the field underwent a notable paradigm shift. The core term “nanoconjugates” exhibited the strongest burst intensity (Strength = 2.02), clearly indicating its central role in research focus. Concurrently, the emergence of “cellular uptake” (Strength = 1.38) and “biodistribution” (Strength = 1.12) suggests that research emphasis had deepened to investigate the mechanisms of interaction between these conjugate systems and biological organisms, as well as their in vivo fate. In recent years (2020–present), research has demonstrated a strong application-oriented and refinement trend. Specifically, the emergence of “chitosan nanoparticles” (Strength = 1.75) and “gold nanoparticles” (Strength = 1.71) as key delivery systems highlights that constructing composite functionalized delivery platforms using chitosan and inorganic nanoparticles (such as gold) represents a major current research direction. Meanwhile, the sustained activity of keywords such as “drug delivery” (Strength = 1.11), “antimicrobial activity” (Strength = 1.88), and the more recent “anticancer” (Strength = 1.28) strongly evidences extensive exploration of these chitosan-based nanoconjugate systems in antimicrobial and anticancer therapeutic applications. The latest frontier developments (2023–2025) are marked by the emergence of the keyword “bioavailability” (Strength = 1.13), indicating that research in the field is advancing beyond simple preparation and in vitro validation toward in-depth optimization of pharmacokinetic properties, ultimately aiming to enhance clinical translation potential. The evolution of infected wound healing with chitosan-based wound care, as revealed by keyword timezone mapping, chitosan-based hydrogel can be categorized into distinct directions: Functional Chitosan-based Hydrogel and Chitosan-based Delivery System.

### 4.1. Functional chitosan-based hydrogel

Each year, thousands of patients suffer from various forms of epidermal damage or burns caused by hot water, flames, accidents, and boiling oil. Winter’s pioneering work in 1977 introduced the 1st generation of wound films or “dressings,” demonstrating that the epithelial repair of wounded pig skin was at least twice as fast compared to wounds exposed to air.^[17]^ Since then, research and development in wound dressings have advanced significantly.^[18,19]^

Chitosan, a polymer derived from chitin, is soluble in acidic aqueous solutions due to its degree of acetylation. This degree can be expressed as a percentage of acetylation or as the molar fraction of N-acetylated units. When the DA is below 50% (0.5), chitosan becomes soluble due to the protonation of the amino group at the C-2 position of glucosamine units. The chitosan-based hydrogels were found to be biodegradable and pH-sensitive, with a maximum swelling degree of 10.220%. The results indicated that these hydrogels hold significant potential as scaffolds for burn wound management.^[20]^ The mechanism by which chitosan-based hydrogels aid in infected wound healing involves binding to bacterial DNA due to the negative charge on the bacterial surface. Additionally, chitosan can interact with the plasma membrane of bacteria, further enhancing its therapeutic effects.

Treating wound infections becomes more challenging in the presence of a biofilm, which acts as a protective barrier for bacteria and greatly reduces the efficacy of conventional antibiotics. This issue is further compounded by the rising global prevalence of drug-resistant bacterial strains, making biofilm-associated infections a critical clinical concern worldwide. Chitosan possesses several unique properties that make it an attractive material for the development of wound dressings. Besides antibacterial function, functional chitosan-based hydrogel has adhensive,^[21,22]^ hemostatic,^[23]^ self-healing,^[24]^ and anti-oxidative^[25]^ functions.

Injectable self-healing hydrogel dressings with multifunctional properties, such as anti-infection, anti-oxidation, and conductivity to promote wound healing, are highly sought after for wound care applications, yet their design remains a challenge. We developed a series of injectable conductive self-healing hydrogels based on quaternized chitosan-g-polyaniline and benzaldehyde-functionalized poly(ethylene glycol)-co-poly(glycerol sebacate) (PEGS-FA). These hydrogels serve as antibacterial, antioxidant, and electro-active dressings for enhancing cutaneous wound healing.^[26]^ By combining the dynamic Schiff base and copolymer micelle cross-linking in 1 system, a series of hydrogels were prepared by mixing quaternized chitosan (QCS) and benzaldehyde-terminated PluronicF127 (PF127-CHO) under physiological conditions. The antibacterial properties, pH-dependent biodegradation, and release behavior of chitosan-based hydrogels were explored to confirm their multifunctional potential as wound dressings.^[27]^ Baolin Guo and colleagues developed antibacterial, adhesive, antioxidant, and conductive grafted-dopamine/chitosan/carbon nanotubes composite hydrogels to improve the healing of infected wounds.^[25]^ Additionally, the research group designed a smart bioadhesive hydrogel made of ferric iron, protocatechualdehyde containing catechol and aldehyde groups, and QCS, which promotes the closure of skin incisions and enhances healing in methicillin-resistant *S aureus*-infected wounds. The results demonstrated that the hydrogel is injectable, biocompatible, and exhibits antibacterial activity, multifunctional adhesiveness, hemostasis, and near-infrared responsiveness. In vivo studies using rat skin incision and infected full-thickness skin wound models showed high wound closure efficacy and effective post-closure care.^[28]^ To date, functional chitosan-based hydrogels have shown great potential in treating skin incisions and infected full-thickness skin wounds.

### 4.2. Chitosan-based delivery system

Chitosan is widely considered a valuable component in delivery systems due to its inherent antimicrobial, anti-inflammatory, antioxidant, and wound healing properties. As one of the most commonly used polymers in such systems, sometimes 3-diamtional printed, it has been reported that chitosan-containing and chitosan-coated liposomes designed for infected wounds enhance the antimicrobial activity of the membrane-active agent chlorhexidine, while maintaining anti-inflammatory effects and cell compatibility.^[29,30]^ Chitosan-based hydrogels containing silver nanoparticles demonstrated the highest activity against resistant bacteria isolated from diabetic foot infections. This composite effectively prevented foot infections caused by multidrug-resistant bacteria and significantly accelerated wound healing.^[31]^ Nano Ag/zinc oxide (ZnO) hybrid materials are regarded as promising nanocomposites for biomedical applications due to their enhanced antibacterial activity and low cytotoxicity. In this study, a sponge-like nano Ag/ZnO-loaded chitosan composite dressing was synthesized by 1st preparing a chitosan sponge through a lyophilization process, followed by incorporating Ag/ZnO nanocomposites into the sponge. The porosity, swelling, blood clotting, and in vitro antibacterial activity against both drug-sensitive and drug-resistant pathogens were evaluated.^[32]^ Composites of PVA/chitosan/ZnO are reported to have a high antibacterial activity, higher than antibiotics such as erythromycin and Met-ronidazole.^[33]^ Therefore, nanoparticles in chitosan-based hydrogel delivery system show great potential for avoiding infection and enhancing wound healing.

## 5. Conclusion

Infected wound healing remains a significant global challenge. Chitosan-based strategies for infected wounds care represent an increasingly important and clinically relevant option. Bibliometric analysis reveals that China, India, and Iran are the primary contributors to this field. Promising therapies nearing clinical translation include multifunctional hydrogels (e.g., self-healing, conductive and quaternized chitosan-g-polyaniline systems), chitosan/metal oxide nanocomposites (such as Ag/ZnO–chitosan sponges), and smart bioadhesive dressings with stimuli–responsive properties (e.g., near-infrared-activated Fe-QCS hydrogels). Future efforts should focus on developing chitosan-based technologies that not only address current wound infections but also remain effective against emerging drug-resistant bacteria.

## Author contributions

**Conceptualization:** Lan-Jing Zhang.

**Data curation:** Hui-Wen Zhang.

**Investigation:** Zou-Zou Yu, Ying-Ying Liu.

**Methodology:** Dong-Dan Wang, Yue Shang.
